# The Role of Computer Simulation in Nanoporous Metals—A Review

**DOI:** 10.3390/ma8085060

**Published:** 2015-08-07

**Authors:** Re Xia, Run Ni Wu, Yi Lun Liu, Xiao Yu Sun

**Affiliations:** 1 Key Laboratory of Hubei Province for Water Jet Theory and New Technology, Wuhan University, Wuhan 430072, China; E-Mail: wurunni@163.com; 2 State Key Laboratory for Strength and Vibration of Mechanical Structures, School of Aerospace Engineering, Xi’an Jiaotong University, Xi’an 710049, China; E-Mail: yilunliu@mail.xjtu.edu.cn; 3 Department of Engineering Mechanics, School of Civil Engineering, Wuhan University, Wuhan 430072, China

**Keywords:** nanoporous metal, simulation, molecular dynamics, kinetic Monte Carlo, dealloying, coarsening, mechanical properties, thermal conductivity

## Abstract

Nanoporous metals (NPMs) have proven to be all-round candidates in versatile and diverse applications. In this decade, interest has grown in the fabrication, characterization and applications of these intriguing materials. Most existing reviews focus on the experimental and theoretical works rather than the numerical simulation. Actually, with numerous experiments and theory analysis, studies based on computer simulation, which may model complex microstructure in more realistic ways, play a key role in understanding and predicting the behaviors of NPMs. In this review, we present a comprehensive overview of the computer simulations of NPMs, which are prepared through chemical dealloying. Firstly, we summarize the various simulation approaches to preparation, processing, and the basic physical and chemical properties of NPMs. In this part, the emphasis is attached to works involving dealloying, coarsening and mechanical properties. Then, we conclude with the latest progress as well as the future challenges in simulation studies. We believe that highlighting the importance of simulations will help to better understand the properties of novel materials and help with new scientific research on these materials.

## 1. Introduction

Nanoporous materials are known as a kind of three-dimensional (3D) porous solid with nanoscale characteristic size [1]. They exhibit a similar morphology to macroscopic foams, but possess smaller pore or ligament diameter and very high specific surface area [2], therefore entailing both the foam-like optimized structure and functionalized feature of nanomaterials from dual-optimization [3]. Due to the known performance advantages, considerable efforts have been made to explore potential applications of nanoporous materials in various fields, such as catalysis, sensors, actuators, fuel cells, energy storage, bioelectrochemistry, plasmonic metamaterials, *etc.* [4,5,6,7].

Nanoporous metals (NPMs) as a typical branch of nanoporous materials, possess metallic features like high strength, good heat resistance and conductivity, as well as the excellent comprehensive properties of porous solids [8]. Increasing evidence shows the intriguing properties and promising prospects of these materials. Easily tailorable microstructure and ultimate strength give NPMs superior mechanical characteristics [9]. Large and electrically tunable strain is desired for the high-effective actuator [10,11]. Controllable optical properties and ultra-low magnetoresistance enable them to function under extreme conditions [12,13]. NPMs also show extraordinary catalytic activity for CO oxidation and ultrasensitive electrochemical response to biological macromolecule detection [14,15].

**Figure 1:**
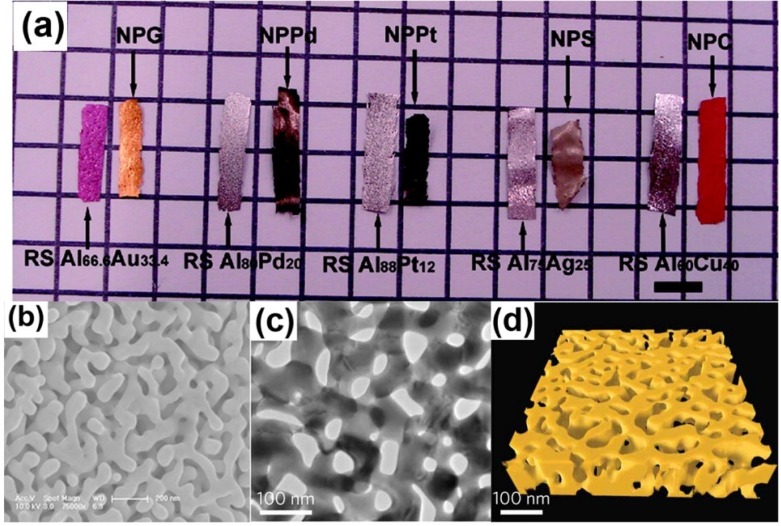
Characterization of nanoporous metals: (**a**) Macrographs of alloys and corresponding nanoporous metals ribbons. Reproduced with permission from Reference [22]. (Copyright American Chemical Society 2009); (**b**) SEM micrographs of NPG; (**c**) TEM micrographs; and (**d**) 3D topographic reconstruction. Reproduced with permission from Reference [23]. (Copyright Nature Publishing Group 2012).

Most previous works on NPMs have been revisited multiple times in recent years, including works on fabrication, characterization and applications [16,17,18,19,20]. With the majority of reviews dedicated to the experimental and theoretical findings, the computational researches have long been overlooked. In fact, the latter has provided an effective solution in illustrating some phenomena which could not be explained by experimental measurement, especially for those controversial issues on NPMs [21]. NPMs made by dealloying show significant microstructure complexity, producing random and bicontinuous open nanoporosity extending in 3D [22,23], as shown in Figure 1. Compared with the idealized geometry used in theoretical studies, to some degree, computer simulation can model more realistic structures and thus provide more reliable results.

For examples, atomistic modeling such as kinetic Monte Carlo (KMC) and molecular dynamics (MD) simulations has been used effectively in the study of structure forming and evolving [24,25], and physical and chemical behaviors of NPMs [26]. The direct experimental observation of the microstructure evolution of NPMs is not currently feasible in most cases. Computer simulations can describe macroscopic properties of NPMs and provide a valid approach to dealloying processes and microstructure evolutions, which are extremely useful to explore coarsening methods, surface diffusion and void formation of NPMs.

The purpose of this review is to give a comprehensive introduction of the current development of simulation study on NPMs. Although there are various approaches to synthesis NPMs, such as the template method and combustion strategy, we focus on the NPMs prepared by chemical dealloying which typically exhibit the 3D-bicontinuous nanoporous structure. In the first part, we briefly summarize the relevant simulation methods widely used in the study of NMPs. A subsequent section is focused on NPM synthesis, especially the corrosion mechanism of the dealloying process. Section 4 encapsulates the research progress in mechanical properties of NPMs, which has attracted an increasing amount of research via computer simulation. Section 5 focuses on other properties of NPMs such as thermal, optical, radiant, *etc.* Outlook on future challenges is then presented following a brief summary.

## 2. Simulation Methods

### 2.1. Ab Initio Calculation

*Ab initio* calculation is a prized and precise computational method, which has been employed to investigate the origin of mechanical, electronic, and magnetic properties of materials and molecules [27,28,29,30]. This calculation is based on the established physics laws of quantum mechanics and does not make any empirical assumptions or parameters fitting [31,32]. For instance, when calculating the electronic structure of atoms by *ab initio* calculations, only Schrödinger’s equation and several approximations are used [33,34]. However, it can provide an accurate approximation of the true state of the system. The most commonly used form of Schrödinger’s equation is the time-dependent Schrödinger’s equation [35]

(1) $$i\hbar \frac{\partial }{\partial t}\Psi =\hat{\text{H}}\Psi$$

where *i* is the imaginary unit, ℏ is the Planck constant divided by 2π Ψ is the wave function of the system, and $\hat{\text{H}}$ is the Hamiltonian operator. The computational scale of *ab initio* calculation is usually small because this method takes larger amounts of computer time. Therefore, *ab initio* calculation is often used for obtaining potentials which will be applied in molecular dynamics simulation, but not directly applied to simulate the mechanical properties for large-scale NPMs [23,36].

### 2.2. Molecular Dynamics

Molecular dynamics (MD) is a computer simulation technique, which can describe the movements of atoms or molecules in a large system to obtain their physical properties, such as the strength, Young’s modulus and flow stress [37,38,39]. In this method, all the atoms are given an initial location and velocities, and then allowed to interact with each other for a period of time. Usually, the coordinates, velocities and accelerations of atoms are determined by solving Newton’s equations of motion numerically for the interacting particles in the system [40]:

(2) $$m_{i}{\ddot{r}}_{i}=f_{i},\;f_{i}=-\frac{\partial }{\partial r_{i}}u_{i}$$

where $m_{i}$, $r_{i}$, $f_{i}$ and $u_{i}$ are the mass, coordinates, forces and potential energy of atom *i*. The forces and potential energy between the particles are determined by force fields. Using MD simulations, the mechanical properties and mechanisms of metal in nanoscale can be properly investigated. Simulated physical phenomena range from fatigue crack propagation [41], dislocation evolution [42,43], and shock-induced plasticity [44,45] to softening of nanotwinned metals [46,47]. Indeed, MD simulation is a useful tool to study the physical and mechanical properties of NPMs which can partly replace the costly experiments. However, the limitations of using MD to study NPMs are also similar to those encountered in studying other materials systems. The spatial and temporal scales in MD simulation are usually much smaller than the experiments.

### 2.3. Kinetic Monte Carlo Simulations

MD simulation can reproduce the results of experiments for pure metals or alloys, but the temporal scale of MD simulation is usually nano-second scale, at which it is sometimes hard to describe the evolution of NPMs, e.g., dealloying, microstructure coarsening. Kinetic Monte Carlo (KMC) simulations give the possibility for quantitative research which may be useful to guide some experiments on surface diffusion [48], surface growth [49,50], vacancy diffusion [51,52] and coarsening of domain evolution [53,54]. In KMC simulations, suppose that there are *N* events may occur which are indexed by the variable *i*, where 0 < *i* ≤ *N*. Considering that for each event *i*, there is a transition rate *k_i_* associated with it. For a single event to happen, the average time is $△t={\left(\overset{N}{\underset{i=1}{\sum }}k_{j}\right)}^{-1}$ in this state. During this time, the probability for a specific event *i* occurred is Pj=kj∑i=1Nkp. All events that may take place are recorded during the KMC simulation [55]. The simulation picks one at random according to its weighted probability and the time step is appropriately increased. At this point, the simulation changes the list of all the possible transitions. A new time step is then carefully calculated and this process repeated after updating all the states. The KMC method has been employed to study the evolution of nanoporosity during dealloying [56]. The sharp negative and positive curvature can also be simulated by KMC simulations [57]. It is noted that KMC sensitively relies on some parameters such as rate catalog. Despite this limitations, KMC is an effective approach to make predictions at the meso scale and it can be used to provide input for finite element simulations.

### 2.4. Finite Element Method

The finite element method (FEM) is also a classical tool to investigate the mechanical properties of NPMs. In FEM, the phenomena are expressed by governing equations and boundary conditions. The structure is cut into small elements, and these elements are reconnected at nodes. Then, the governing equations and boundary conditions of all the elements are approximated as a set of simultaneous algebraic equations:

(3) [K]{u}={F}

where [K] is the properties such as stiffness, conductivity and viscosity, {F} is the action such as force, heat source and body force, and {u} is the unknown behavior such as the displacement, temperature and velocity. By solving these equations, the unknown behaviors are obtained. Stiffening behaviors of porous solids, which possess various configurations and relative densities, have been modeled using FEM [58]. Numerical simulations and finite element analyses have also been carried out to understand the lattice rotation and kinematics of finite deformation of ligaments of NPMs [59], nanoindentation properties and the surface elasticity and initial stress effects of a porous media [60,61]. In order to implement the FEM simulation of NMPs, the mechanical properties (e.g., Young’s modulus, Poisson’s ratio, yield strength, *etc.*) of the ligaments of NMPs must be obtained through experiments or other computation simulations. Due to the inherent errors, FEM only obtains approximate solutions; however, it is still a powerful tool to deal with very complex geometry, loading and restraints for a variety of problems.

### 2.5. Phase Field Method

The establishment of appropriate calculation models with microstructures close to those in real NPMs is important for simulating their mechanical and physical properties. Most NPMs have bicontinuous structures, which consist of open cells with random characteristic sizes [62]. Newman *et al.* [63] have shown that the bicontinuous structures of NPMs are very similar to the morphology of spinodally decomposed materials. The process of spinodal decomposition can be adequately described through phase field method [21,26,64]. In this phase field method, a binary fluid mixture with two components which are uniformly distributed at the initial state, when it undergoes rapid cooling below a critical temperature, will spontaneously decompose into two different phases [64]. Then, one phase is removed to obtain the pores, and the remaining phase becomes a nanoporous microstructure. The evolution of the spinodal structure can be described by the Cahn-Hilliard equation [65]:

(4) $$\frac{\partial u}{\partial t}=\Delta [\frac{\text{d}f(u)}{\text{d}u}-\theta^{2}\Delta u]$$

where u(x,y,z,t) is the difference of the two concentrations (*i.e.*, u∈[−1,1]), f(u) is the homogeneous free energy, and θ is the transition region length between two phases [66]. The phase field method can be used to generate the real bicontinuous structures of NPMs, but the limitation is that the three-dimensional simulation requires significant computer resources.

## 3. Simulation on Alloy Dealloying

Figure 1 displays the typical microstructural characteristics of nanoporous gold (NPG) made by selective dissolution of silver from Ag-Au alloys [22,23]. How to establish a suitable computational model of NPMs is a critical step. Nanoporous materials produced by a dealloying technique often exhibit a bicontinuous microstructure consisting of random-sized open cells. Dealloying, during which an alloy is decomposed by the selective dissolution of the most electro-chemically active elements, is a common corrosion process, which is widely employed to make NPMs in both experiments and simulations. This process usually generates complex 3D-nanoporous morphology of the noble alloy constituents, and the underlying physical mechanisms of the nanoporous structure formation have been studied in recent years.

Indeed, the nanoporous formation during dealloying of alloy can be attributed to an intrinsic dynamical pattern formation procedure process, involving kinetics dissolution, surface diffusion, and mass transport through the bulk of both alloy and electrolyte. Firstly, the processes start with the dissolution of the electro-chemically active atoms on the alloy surface, leaving behind the noble atoms with no lateral coordination (adatoms), then the adatoms diffuse and agglomerate together on the solid-electrolyte interface, known as the spinodal decomposition. As the selective dissolution continues, more noble atoms are chemically driven to gather into noble atom clusters left over from dissolution of previous layers, which ultimately leads to pit formation and porosity. The KMC model is used to simulate the dealloying process of alloys. To take the Au-Ag alloy as an example, this KMC model includes only diffusion of Ag and Au atoms, and dissolution of Ag atoms can reproduce all relevant characteristics of experimental trends (e.g., morphological and kinetic) related to dealloying [24], which shows the dissolution of the electro-chemically active atoms, and surface diffusion and aggregation of the relative noble atoms are the most important procedures to determine the nanoporous structures of NPMs during dealloying.

In order to describe the porosity formation during dealloying of alloy, a continuum mesoscopic model is proposed by accounting for the diffusion of noble atoms on the alloy-electrolyte interface as $\frac{\partial C}{\partial t}=v_{n}C_{0}-v_{n}\text{κ}C-\nabla .J_{s}$, where *C* is the concentration of noble atoms on the alloy-electrolyte interface, *C*_0_ is the initial concentration in the bulk alloy, *v*_n_ is the etching velocity normal to the interface and κ is local curvature of the alloy-electrolyte interface. For *J*_s_, it is the flux of disusing adatoms on the alloy-electrolyte interface, which is described by Cahn-Hilliard diffusion equation as $J=-M\left(C\right)\left(\frac{\partial^{2}f}{\partial c^{2}}\right)\nabla C+2M\left(C\right)w\nabla^{3}C$ and the surface aggregation process inherent in the Cahn-Hilliard form is essential for porosity formation. In this model, the free energy of the system f(C,T) was written as $f=\alpha c\left(1-c\right)+k_{B}T\left[clnc+\left(1-c\right)ln\left(1-c\right)\right]$, where *c* = *C*Ω^2/3^ (Ω is the atomic volume of the noble atom) is the local mole fraction of the noble atoms on the alloy-electrolyte interface, *T* is the absolute temperature and $k_{B}$ is the Boltzmann’s constant. *M*(C) is the mobility of the adatoms which is usually proportional to the surface diffusivity *D*_s_ and given by *M*(*C*) = (*D*_s_/*k*_B_*T*)*c*(1−*c*), and *w* is the gradient energy coefficient. Here, the first term in the right of the Cahn-Hilliard diffusion equation presents the chemical driven force of the spinodal decomposition, and the second term indicates the effect, which damps high frequency fluctuations [24]. The wide range of physical phenomena, such as the formation of porosity, the critical potential, the temperature dependence of the dissolution flux, the steady-state dissolution flux, and composition on dealloying, can be reproduced by the continuum model and KMC model [55,67].

The parting limit (defined as the percentage of reactive element below which dealloying vanishes) is another important characteristic during dealloying. Indeed, the parting limit is not sensitive to experimental conditions such as applied potential and is intrinsically geometric in nature. KMC model simulation is used to study the parting limit of dealloying and the results are comparable to the high-density site percolation calculations, which shows that the geometrical connectivity of reactive atoms is the major means to determine the parting limit of dealloying. For example, the parting limit of Au-Ag alloy through KMC model simulation is 54.7% ± 0.3% in accordance with the previous experimental results, but it is slightly larger than the prediction of high-density site percolation thresholds for an FCC lattice (59.97% ± 0.03%) for the reason that a few otherwise inaccessible dissolution paths are opened up by surface diffusion of Au [68]. However, the dealloying process is very complicated and for a few alloy systems such as the Cu-Zn or Cu-Al alloys, the parting limit is about 20%. The possible reasons may lie on the fact that the extreme reactivity of Al enables it to dissolve from highly occluded geometries or the exchange of the Cu atoms in the alloys and the Cu ions in the electrolyte enables the dissolution of the Zn atoms passivated by Cu atoms.

**Figure 2:**
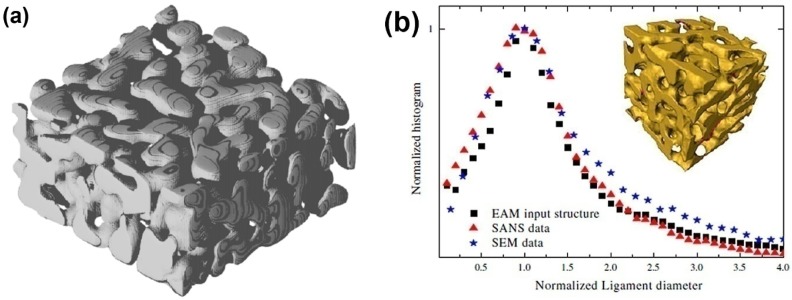
Bicontinuous structure of nanoporous gold: (**a**) Generated by a Monte Carlo model. Reproduced with permission from Reference [56]. (Copyright Elsevier 2013). (**b**) The ligament diameter distribution of the experimental data. Reproduced with permission from Reference [26]. (Copyright Elsevier 2009).

The dealloying process leading to the formation of NPG has also been directly simulated by Metropolis Monte Carlo (MMC) simulation, which adopts a lattice-gas model for Ag, Au vacancies, acid particles, and other products of chemical reactions. This simulation focuses on the influence of the concentration of solute, the temperature of the system and lattice defects on the porosity formation. A minimal three-parameter model, namely the interaction constants of Ag-Ag, Au-Au and Au-Ag, is sufficient to yield NPG structures which have morphological properties akin to those found in the experiment, such as the Euler characteristic, volume, surface area and the specific surface area [56], as shown in Figure 2a. The ligament size of dealloyed material is on the order of 2–3 nm, which is comparable to the experimental data as shown in Figure 2b [26]. However, for the very small size of the ligaments, the surface stress will drive the mechanical instability of the ligaments so that a large volume change is observed [26], namely the coarsening effect, which will be presented in detail in next section.

Besides the dealloying approach, there is another method, namely the expanding lattice approach, to generate NPMs. The expanding lattice approach begins with building a crystalline supercell with a given number of atoms and a density which is close to the solid materials; then the nanoporous materials are generated by rapidly increasing the volume of the cell at a high temperature near the melt point of the solid materials. This expanding lattice approach is successfully applied to generate nanoporous structures of semiconducting (carbon, silicon) and metallic (copper, silver) materials [69]. However, when comparing the pair distribution functions of the NPMs generated by the expanding lattice approach and dealloying approach with the experimental results, the dealloying technique is more suitable when dealing with alloy systems. For the dealloying approach, the effects of the etching time, electrolyte concentration, and pre/post heat treatment on the morphology of the NPM have been investigated. By using the Design Expert 7.1 software, a model relating the processing parameters and pore size is established using statistical approaches [70].

In addition, Monte Carlo simulations predicted that it is possible to form two porous networks with nearly equal packing density through the self-assembly of one building block, which is confirmed by the scanning tunneling microscopy (STM)-based experiments [71,72]. The final structure of two nanoporous networks can be tuned by controlling the solvent, concentration and temperature during pattern formation, and utilized as host networks to trap guest molecules or guest complexes selectively.

## 4. Mechanism of Coarsening

**Figure 3:**
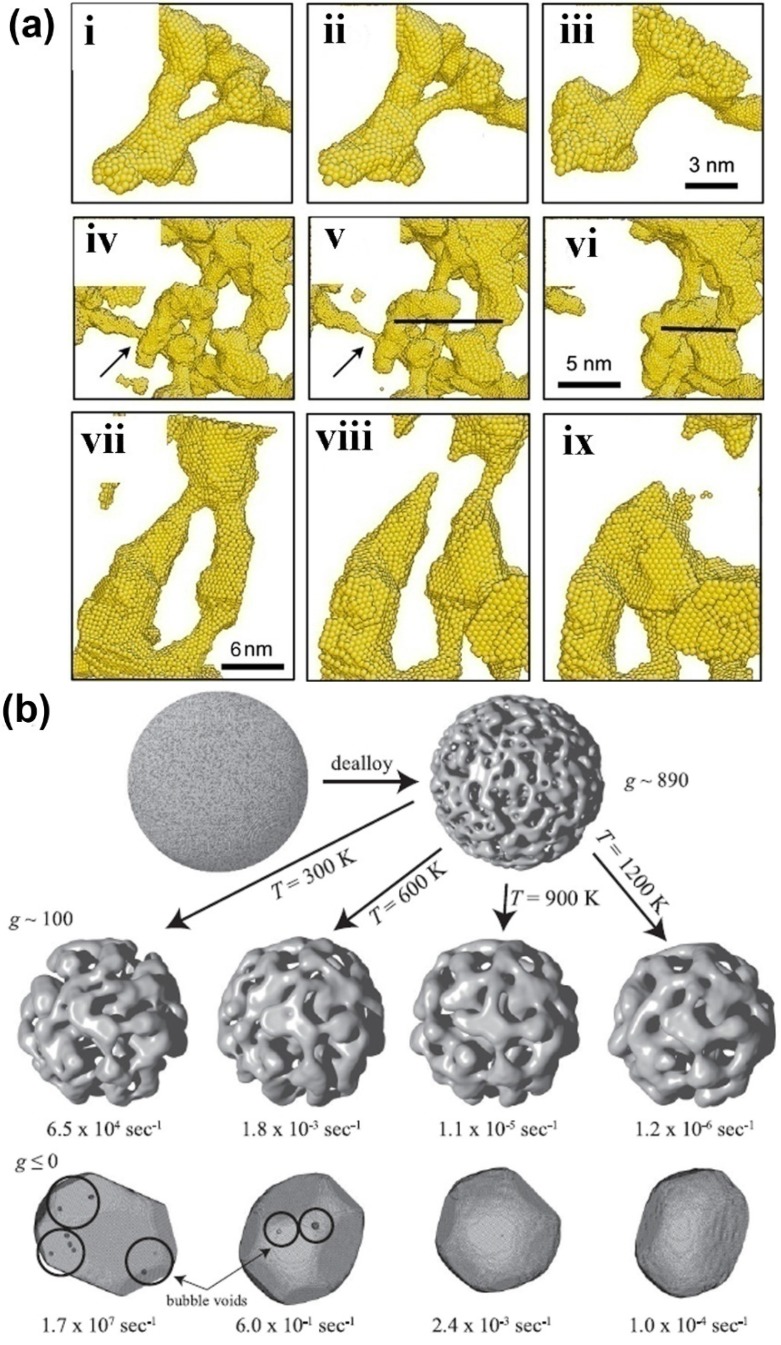
(**a**) Several examples of ligament collapse during annealing at constant volume; in (i–iii) the collapse to two neighboring ligaments, (iv–vi) the pinch-off of the ligament marked by an arrow and (vii–ix) the collapse of two pinched-off ligaments. Reproduced with permission from Reference [73]. (Copyright Elsevier 2011). (**b**) Snapshots of microstructure evolution of NPMs from high topological genus nanoporous nanoparticle to Wulff shape at different temperatures. Reproduced with permission from Reference [74]. (Copyright American Physical Society 2011).

The coarsening of NPMs is one of the most important microstructure evolutions, which is usually observed in annealing and dealloying processes of NPMs. Currently, there are only limited simulation works to study the coarsening mechanism of NPMs [57,73,74]. Two mechanisms are proposed, *i.e.*, localized plastic deformation-controlled collapse of the neighboring ligaments [73] and the ligament pinch-off or bubble formation caused by surface diffusion-controlled solid-state Rayleigh instability of the ligaments [74]. For the collapse of neighboring ligaments, the neighboring ligaments usually adhere to one point. Then, with the collapse proceeding, the neighboring ligaments gradually become one large ligament, as in Figure 3a. During the collapse, the ligaments are bending, so that large plastic deformation is induced in the ligaments, which may prevent the development of the ligament collapse. As for the solid-state Rayleigh instability of ligaments, it is similar to the liquid Rayleigh instability, where the liquid cylinder spontaneously changes to a row of droplets. During the coarsening of NPMs, the ligaments first become thinner and then pinch off so that the topological genus of NPMs decreases during annealing and dealloying (see Figure 3a,b).

The collapse of the neighboring ligaments is determined by the competition between the plastic work required to bend the ligaments and the reduction of the surface energy through the reduction of the free surface of the NPMs. As shown in the atomistic simulations, coarsening stops when the radius of the interconnected ligaments increases to a certain value, which is dependent on the relative density and pore size of the NPMs. Hence, the critical ligament radius for spontaneous ligaments collapse can be estimated through the balance between the surface energy reduced and the plastic work required, that is, $R^{*}=K\frac{\gamma }{\text{τ}}$, where $K=K_{0}\left(C_{0}-2\frac{p_{2}}{p_{1}}C_{2}^{'}\sqrt{{\text{ρ}}_{\text{rel}}}\right)$, where γ is the surface energy, τ is the flow resistance of the parent materials of the NPMs, *C*_0_ is a measure of the extent of collapse of ligaments onto each other, *K*_0_ is the ratio between the diameter of the pore and the displacement of a ligament during collapse of the neighboring ligaments (usually in the order of 1), ρ_rel_ is the relative density of the NPMs, *p*_1_ is the collapsed fraction of the ligaments and *p*_2_ is the pinch-off ligaments that do not collapse. The detailed derivation of the critical radius of the ligaments can be seen in Reference [73].

In contrast, surface diffusion-controlled solid-state Rayleigh instability controls both bubble formation and ligament pinch-off, so that high topological genus NPMs evolve toward the Wulff shape (see Figure 3b) [74]. Indeed, the kinetics of ligament pinch-off are controlled by the Rayleigh instabilities of the ligaments. The typical Rayleigh instability problem analyzes a cylinder of radius *R*_0_, whose radius is perturbed by the instability of wavelength. For wavelengths longer than a critical λ_c_ the perturbation will grow because the total surface energy of the system decreases and ultimately, the cylinder breaks up into a series of droplets. As the Rayleigh instability is determined by the surface energy only, if we consider a cylindrical void, the void space should break up into series bubbles, which is bubble formation. The mass transport of the ligaments in Rayleigh instability is controlled by surface atom diffusion. The diffusion time constant for an unstable mode to grow is given by τ_λc_ ~ λ^4^, where τ_λc_ is the critical time constant for wave-length λ. The reason for bubbles being formed first is that all characteristic lengths are small. Once formed, these bubbles are stagnant because the characteristic length of the structure is always larger than any void. Ligaments, in contrast, are never stable, and ligament pinch-off can destroy nearby bubbles; thus, over a longer time the number of bubbles decreases [74].

## 5. Mechanical Behavior of Nanoporous Metals

### 5.1. Surface Effect on Mechanical Properties

The surface-to-mass ratio of NPMs is in the range of 100–1000 m^2^/g, an order of magnitude higher than that of porous materials in microscale [75]. The change of surface state will significantly affect the mechanical properties of nanoporous materials. For example, due to the surface stress, volume of the NPM decreased when one constituent was removed from the alloy by selective dissolution to create the bicontinuous NPMs [76]. In order to check the dominant mechanisms for the geometric relaxation of NPMs, a phase-field model describing the spinodal decomposition process of a binary alloy has been conducted [77]. The Cahn–Hilliard equation $\frac{\partial C}{\partial t}=-\nabla^{2}\left(F\left(C\right)+\nabla^{2}C\right)$ can be used to describe the evolution of the spinodal morphology, where *C* is the local composition and *F* is the local free density potential. Then, created nanoporous samples were relaxed by molecular simulations which can perform isotropic relaxation of the sample. Geometric volume variation of nanoporous structures has been shown to be significantly dependent on surface relaxation rather than the curvature-dependent mean pressure.

Surface relaxation decreases the geometric volume and affects the mechanical stability of NPMs with small ligament sizes. To understand the stability of the nanoporous structures, MD simulations of NPG have been conducted [26]. The surface stress is set in the range of 1.7–2.6 Jm^−2^ and the ligament size varies from 1.3 to 3.6 nm. Simulations indicate that spontaneous plasticity of NPMs results from the surface stress. Under different surface stress, the plasticity which is induced by surface stress can take place with different surface-volume ratios.

The variation of surface stress can change the volume of NPMs, which shows the possibility of a high-performance surface-chemistry-driven actuator [21]. The changes induced by surface chemistry of the surface stress of NPMs lead to an elastic mechanical response, which has been verified by MD simulations. The bicontinuous network of NPG model was generated by simulating the spinodal decomposition. After achieving the desired ligament size, this process was frozen. The samples were equilibrated at various temperatures ranging from 0 to 300 K. During this relaxation, the dimensional changes resulted from tensile surface stress. It is found that the relationship between the average change of surface stress ˂∆f˃ and the dimensional changeis $˂∆f˃=-9k/\left(2{\text{α}}_{\text{m}}\rho \right)\left(∆l/l_{0}\right)$, where *k* is the bulk modulus of the solid, ${\text{α}}_{\text{m}}$ is the specific surface area, ${\text{α}}_{\text{m}}$ and ρ is the bulk density [21]. The relative changes were also investigated including the volume of the solid, pore volume and total sample volume for the deformation of a porous solid in response to the capillary forces on the pore surfaces [78]. The deformation strains are imposed when the pore size is in the range of nanometers.

Plating thin layers of metal atoms onto an NPG can increase the strength and hardness but decreases creep deformation. A MD model of core-shell nanowires has been used to explore the differences in the deformation mechanisms with the addition of the plated Ni [79]. Twinning plays a larger role in a core-shell structure, whereas a comparable size structure in a monolithic material is primarily dominated by the nucleation of dislocations. The use of atomistic simulations in conjunction with conventional models of the strengths of foams shows that the predicted performance of the foam using the simulation is within 15% of the experimental data for relatively low-density foams.

### 5.2. Deformation Mechanisms under Uniaxial Tension/Compression Loading

Many efforts have been devoted to explore the deformation mechanisms of NPMs under uniaxial tension and compression loading. Using a tetrakaidecahedron simulation model, the mechanical behaviors of closed-cell nanoporous copper foams can be investigated [80]. A tetrakaidecahedron is a fourteen-sided figure composed of six squares and eight regular hexagons, and it is formed when six square pyramids are removed from the corners of a regular octahedron. Closed-cell nanoporous foams show close agreement between the simulated elastic moduli and constitutive theory. However, the open-cell foam shows a fluid-like property because the struts are so small that the open-cell foam in this model is semi-stable.

MD has been performed to quantify the evolution of dislocations and the configurations in a porous single-crystal metal. In the simulations, the average initial porosity is set at 4.1%, and the radius of porous metal is about 3.3 nm [81]. Simulations show that nanovoids are effective dislocation sources. During the uniaxial compression, the dislocation shear loops nucleate at the nanovoid surface. Nanovoids can lead to an elastoplastic response, which is unlike that of single crystal. MD simulations have shown that plastic deformation proceeds by the dislocation emission from the void surfaces and by dislocation propagation in the ligaments and dislocation interaction when they meet from adjacent voids. As voids start to shrink, the dislocation density increases rapidly [82].

The mechanical deformation of NPMs under high strain rate loading is examined by performing MD simulations [83]. Due to the large plastic deformation resulting from nanovoids, which act as the dislocation sources, nanosacle grains are formed in single-crystal nanoporous copper samples. Phenomena including large dislocation densities near the nanovoids, the formation of partial dislocation loops and extended dislocation networks are observed. The strength of the nanoporous material is changed by various dislocation structures, which are induced by the void-void interactions and collective processes.

The softening behaviors of nanoporous aluminum under tension have also been examined by MD simulations [84]. Nanoporous models have been generated by creating randomly spheres, which overlap with each other. Then, the atoms in the spheres are deleted to obtain the final atomistic model. The relative density and the characteristic length can be adjusted by changing the radii and separation distance between the spheres. The simulation results show that if the characteristic length of the nanoporous structure is small enough, the relatively compliant ligaments and joints act to spread stress out, and therefore the softening of NPM is observed to slow down significantly [85].

Using a similar random overlapping spheres model, the compressive behavior of nanoporous gold, which has various porosities and ligaments in the range of 0.5–16 nm, has been studied via MD simulations [86]. Three characteristic stages, *i.e.*, the linear elastic region, work hardening region, and densification region were shown when NPG was under a uniaxial compressive loading. The dislocation defects were identified at different strain levels, and the strain hardening behavior was attributed to defects that accumulated at the joints, which connected ligaments in the NPG structures. MD simulations have shown that the dominant deformation mechanism of NPG is ligament bending at the joints of the structure, which is consistent with experimental results on NPG under compression.

**Figure 4:**
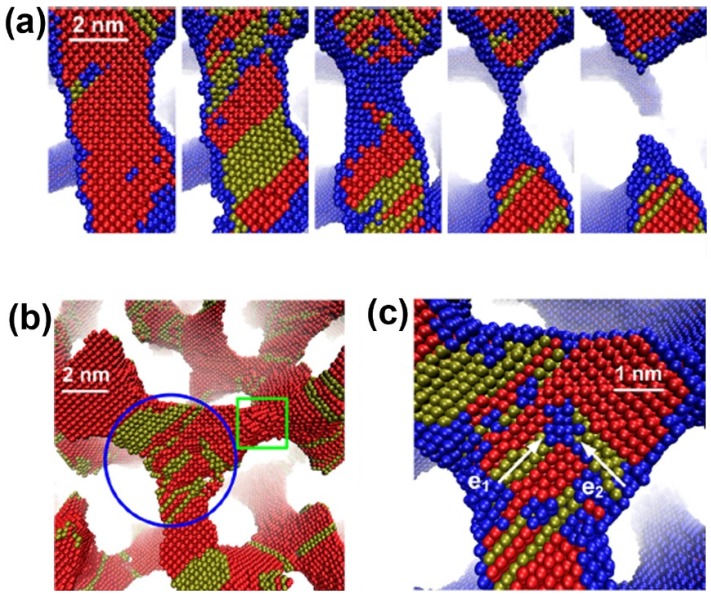
Deformation mechanism of nanoporous gold under uniaxial tension: (**a**) rupture of a ligament; (**b**) dislocation nucleation; and (**c**) a dislocation lock formation in a junction. Reproduced with permission from Reference [87]. (Copyright AIP Publishing LLC 2013).

A phase field simulation was used to create the bicontinuous nanoporous microstructure. MD simulations then are performed to investigate the deformation behaviors and underlying physical mechanisms of NPG under uniaxial tension [87]. Tensile strain leads to progressive necking and rupture of some ligaments (Figure 4a), ultimately resulting in failure of the material. The stacking faults will accumulate in the ligaments along the loading direction and their junctions with other ligaments, and Lomer-Cottrell dislocation locks can form at the junctions, as shown in Figure 4b,c.

MD simulations for tensile and compressive tests of bicontinuous NPG show a significant tension/compression asymmetry [88]. The tension/compression asymmetry can be explained by a model with the surface stress effects on the mechanical response of ligaments as a parameter. This model indicates that the tension/compression asymmetry will take place if the ligament diameters are less than 15–20 nm. The plasticity of NPMs under compaction is in direct proportion to the decrease of the average ligament size and the collapse of the nanopores.

### 5.3. Scaling Laws

Based on the beam theory, Gibson and Ashby derived the expression $E_{\text{s}}/E_{\text{b}}\propto {\text{ρ}}^{2}$ when the ligaments of cellular structures are subjected to bending, where $E_{\text{b}}$ is the Young’s modulus of the bulk phase, and ρ is the relative density. By performing MD simulations, the relationship between the effective Young’s modulus and the relative mass density of NPMs is modified as $\frac{E_{\text{s}}}{E_{\text{b}}}\propto C_{1}\left({\text{ρ}}^{2}+C_{2}\text{ρ}\right)$ [87]. The two terms on the right-hand side correspond mainly to the bending and tensile deformations of ligaments, respectively. The ultimate strength of NPG shows a strong dependence on the average ligament diameter *d*, with an exponential relationship that ${\text{σ}}_{\text{u}}/{\text{σ}}_{\text{b}}\propto d^{-0.44}$.

**Figure 5:**
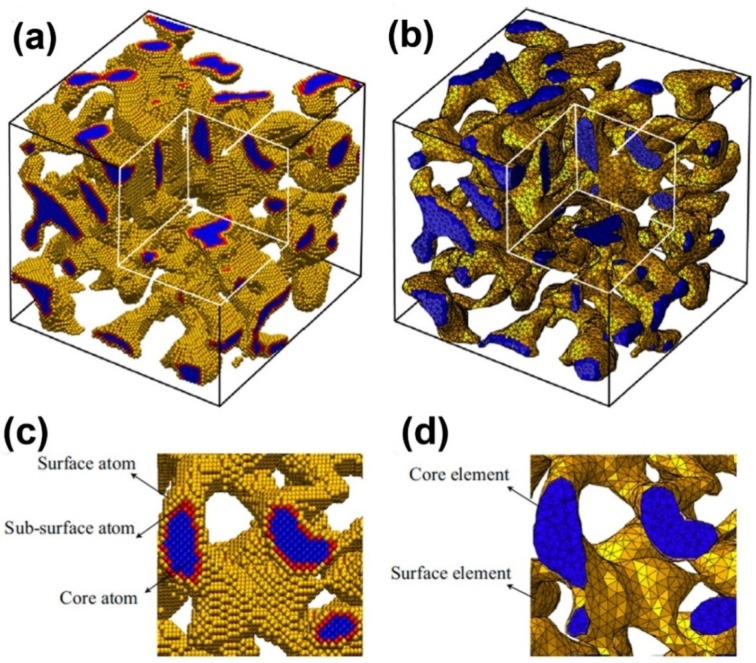
Multi-scale model of nanoporous gold: (**a**) atomistic model and (**b**) continuum model; (**c**) and (**d**) are the enlarged views of a cross-section of (**a**) and (**b**), respectively. Reproduced with permission from Reference [36]. (Copyright Elsevier 2014).

Nanoporous samples are only within a certain range of ligament diameters (2–4 nm) by using MD simulation, because excessive atoms will be contained in simulation model when the ligament diameter is larger. In experiments, the ligament diameter of NPG often varies in the broad range from 2 to 500 nm [89]. Therefore, a multi-scale method, combining the MD simulations and finite element method, is necessary to deal with this problem. Using a multi-scale modeling approach, the charge-induced deformation, stiffness and plastic strength of three NPG structures (cubic lattices, gyroids and disordered) as a function of relative density, surface-to-volume ratio and crystal orientation were investigated (Figure 5) [36]. An atomistic model, which is calibrated to density functional theory, was developed to account for the excess charge, and a surface layer model which is informed by atomistic simulations is proposed to make a scale transition from the atomistic to the continuum scale. The effective Young’s modulus and actuation strain of nanoporous structures can be written as ${\text{ε}}_{\text{c}}=c_{3}{\left(V_{\text{s}}/A_{\text{s}}\text{α}\right)}^{\text{γ}}$, where $\text{α},\text{ β},\text{ γ},\;C_{1},\;C_{2},C_{3}$ are scaling parameters, as shown in Table I of Reference [36].

## 6. Other Properties

### 6.1. Thermal Properties

Thermal responses are among the most important features of metal foam on their optical and electronic applications. For NPMs, the related studies currently concentrate on two aspects: measurement of basic material parameters like thermal conductivity [90], expansion coefficient and Debye temperature [91], and effect of various treatments on thermal properties like the effect of annealing or alloying on microstructure change and thermal conductivity [92,93,94]. In this part, the recent advances in the simulation works on thermal response of NPMs are revisited.

By 2004, using the Lennard–Jones argon MD model, the effects of different temperatures and pore configurations on thermal transport of nanoporous thin films have been simulated [95]. Thermal conductivity of thin films decreases as porosity and simulation temperature grows, while the influence of pore shape is negligible. Significantly, the position of pore in films is crucial as placing the pore in the center leads to a lowest thermal conductivity. Compared to that of bulk, nanoporous Si exhibits two orders of magnitude reduction in transverse thermal conductivity (the conductivity in the plane perpendicular to the cylindrical pore axis), which are likely to result in the reduction in the channels for phonon transport and increasing phonon scattering at the pore surfaces due to the increasing pore volume and surface-to-volume ratio, respectively.

Through Monte Carlo simulation, nanoporous alloys were found to exhibit thermal conductivity reductions with larger pore sizes, which is essentially different from the non-alloyed porous materials [96]. Even containing relatively larger pores, the thermal conductivity of alloy exhibits an obvious decrease, but a similar trend occurs only for the pure materials with nanoscale pores. For example, porous Si_0.5_Ge_0.5_ alloy (0.1 porosity) can reach the same relative reduction (50%) in thermal conductivity as pure Si or Ge, with tenfold larger pore diameters (1 μm). The finding suggests that nanoporous alloys are more competitive in terms of thermoelectric functions.

### 6.2. Optical and Magnetic Properties

Surface-enhanced Raman scattering (SERS) and surface plasmon resonance (SPR) are the most frequently studied features of the optical behavior of NPMs [97,98,99,100]. Corresponding works have concerned the dependence of SERS and SPR on the composition, geometry and characteristic size of samples, which provokes the functions of NPMs in biological and chemical sensing.

A combination of experimental electron energy loss spectroscopy (EELS) and discrete dipole scattering (DDSCAT) approach is effective in exploring the nano-scale polychromaticity of NPMs, where the DDSCAT simulations provide a more comprehensive view of the local field enhancements to make up for the lack of three-dimensionality in EELS maps [101]. It is found that the plasmonic resonance energy strongly depends on the *in situ* surface characteristics at the location such as size and shape of the surface protrusions. These easily tunable optical absorption characteristics further promote the application of NPMs in biosensing field [102,103]. The finite difference time domain (FDTD) simulation could be used to explore the plasmonic features of NPMs [104]. By comparing the electric field distribution of NPG disk to Au disk in the identical shape and size, the origin of plasmonic coupling is verified so that random nanoporous structure promotes Plasmon-matter interactions and the special shape would enhance the effective plasmonic resonance of NPG disks.

MD simulations and first-principles calculations, based on the generalized EAM potential, are in a position to examine the magnetic properties NPMs [105]. The electrodeposits of Co atoms may change the magnetic behavior of NPG obviously. Large lattice contraction gives rise to the low magnetic saturation, but the high coercivity of Co deposited NPG is the results of the surface spin disorder and the larger lattice strain.

### 6.3. High Catalytic Activity and Radiation Tolerance

**Figure 6:**
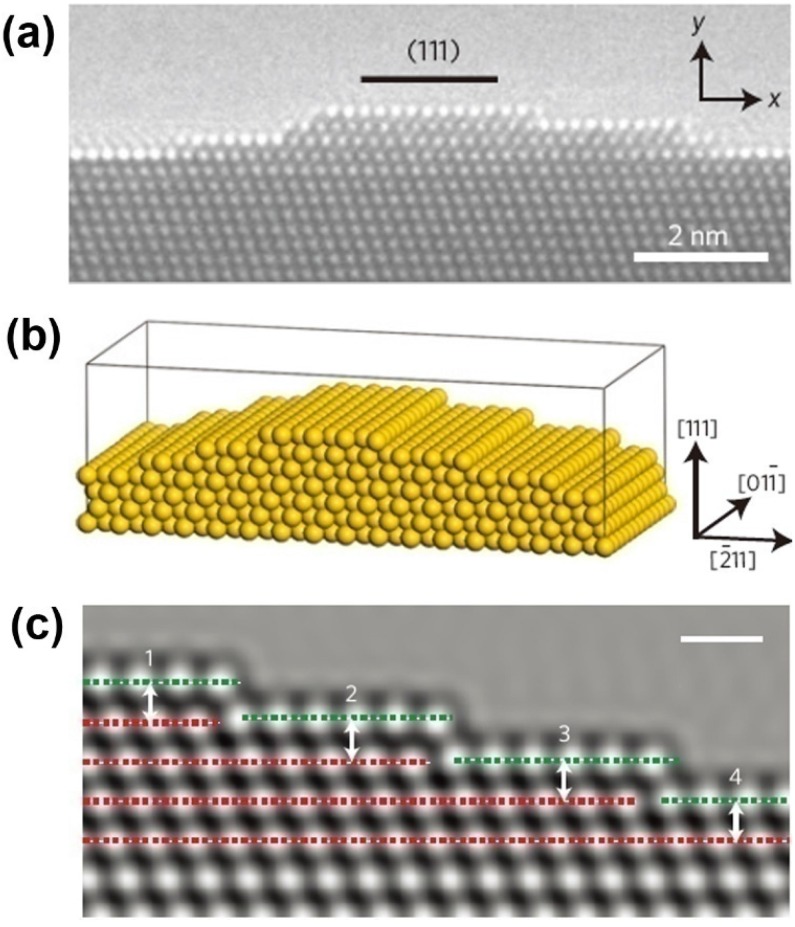
Characterization and simulation model of NPG surface strain: (**a**) HRTEM micrograph of a convex surface of NPG; (**b**) Structural model with a (111) surface plane; and (**c**) Simulated HRTEM image of surface configuration to evaluate the surface strain. Reproduced with permission from Reference [23]. (Copyright Nature Publishing Group 2012).

Due to remarkable catalytic activity and structure-sensitive catalytic properties, along with the advantage of self-supporting, and thermal and mechanical stability, NPMs have been highlighted as promising materials for catalytic applications [106]. Even with the larger ligament size (>30 nm) or at temperatures down to −30 °C, NPG still retains remarkable catalytic activity for CO oxidation at ambient pressures [23,107]. In the discussion of atomic origins of NPG catalysis, *ab initio* simulation has been performed to determine the cause of the surface strain [23]. It has been found that the lattice defects exert great influence on the surface strain. This finding agrees well with the observations of spherical-aberration-corrected transmission electron microscopy (TEM) and environmental TEM (Figure 6).

Perfect radiation tolerance is a notable advantage for NPMs [108]. The potential associations between irradiation response of NPG and its characteristic size have been revealed by using MD analysis [109]. The results show that only when the ligament size is reasonably large would NPG behave as bulk materials under irradiation. Critical size has been denoted as the distance that the defects migrate in the time interval between collision cascades. The MD simulations of defect formation in ligaments during radiation damage also demonstrated the importance of ligament size to radiation tolerance of NPMs [110]. High specific surface area gives the NPMs substantially different response to radiation damage that happens to bulk materials. Coarsening, stacking fault tetrahedra (SFT) and twinning become the main ways of damaging NPMs. Also, the superior radiation tolerance of nanoporous Ag has been identified based on the experimental observation of the influence of a free surface on defect removal and migration kinetics in irradiated samples [111]. In the process of analyzing *in situ* irradiation experiments in the transmission electron microscope, a group of simulation programs named SRIM were used to calculate the stopping and range of ions into irradiated nanoporous Ag. Obtained depth profiles of radiation damage and Kr ion concentration are indispensable to the evaluation of the radiation damage level under different radiation conditions.

## 7. Summary and Future Prospects

### 7.1. Summary

Due to the complex 3D morphology and random pore/ligament distribution, experimental and theoretical works on NPMs have met with great difficulties and controversies. However, computer simulations are valuable for revealing the formation mechanism and fundamental properties of NPMs. The main focus of current efforts concerning simulations are the kinetics of alloy dealloying during NPMs formation, the mechanism of pore/ligament coarsening occurring in dealloying and thermal treatment, and the extraction of enhanced physical and chemical properties for promising applications, especially mechanical ones, *etc.*

The morphological and kinetic of bicontinuous random nanoporous structures could be well described by KMC simulations, which have been used to reproduce a wide range of physical phenomena including the formation of porosity, the critical potential, and the temperature dependence of the dissolution flux.

The mechanical properties of NPMs are closely correlated with their microstructures (e.g., size, geometry, topology of the ligands and voids). Atomic simulations such as KMC and MD simulations are powerful tools to observe the microstructure evolution during dealloying, annealing and plastic deformation that cannot be observed *in situ* experimentally. The coarsening effect is the most common microstructure evaluation. In general, two mechanisms are suggested to explain the coarsening of the ligaments and bubble formation: the surface diffusion-controlled ligament pinch-off and localized plastic deformation-controlled ligament collapse. The surface diffusion-controlled ligament pinch-off usually occurs at relative high temperature where the thermal energy is large enough to activate the surface diffusion. The driven force is the reduction of the surface energy, but the localized plastic deformation-controlled ligamentcollapse occurs between neighboring ligaments, which is determined by the competition between the surface energy and localized plastic energy.

NPMs possess very high surface area, the change of which can influence their mechanical properties remarkably. The surface effect such as geometric volume variation under surface relaxation, surface-chemistry-induced deformation and surface decorating have been investigated through KMC and MD. Deformation mechanisms of NPMs under uniaxial tension and compression loading are also explored by simulations, and the scaling laws of mechanical properties have been provided.

Other than the mechanical behaviors, advantages of NPMs in thermal, optical and magnetic fields also have been established using computer simulations. The analysis results of thermal conductivity have proved that NPMs (nanoporous alloy) should be among popular candidates for thermoelectric applications. Perfect abilities including SERS, SPR and radiation endurance, and their relations with microstructure characteristics of samples have been verified by different simulation methods, respectively, therefore expanding our understanding of the astonishing versatility of NPMs. Finally, based on the atomic simulation, a better interpretation of atomic origins of the high catalytic activity of NPMs has been elaborated so that we can further develop NPMs with more appealing structural stability and chemical activity.

### 7.2. Future

Looking forward to future studies, a number of challenges remain in the investigation of NPMs, and we mainly list those related to the mechanical analysis of computer simulations:

(1) The mechanical properties of NPMs can be significantly regulated through depositing only an atomic-layer-thick coating on its surface [112]. However, the underlying physical mechanisms are still open to further investigations. Thus, studying the surface modification effects of NPMs on mechanical properties by using multi-scale method is necessary.
(2) The scaling laws for the mechanical properties of NPMs with surface adsorption/modification effects in terms of microstructure and coating parameters need to be developed. Efforts to explore the electric control of surface effects would be useful. In experiments, polycrystalline structural ligaments of NPMs are sometimes observed [113]. However, the ligaments in previous simulations are mostly considered to be monocrystal. It is necessary to find out the mechanical properties of NPMs, which are more sensitive to the ligament size or grain size.
(3) Porous materials are widely used for energy absorption. Under compression, the energy is absorbed by the bending, buckling and fracture of the ligaments of the NPMs. The shock will induce dislocation nucleation in the NPMs. Therefore, the mechanical behavior of NPM under ultrahigh loading rates should be investigated.
(4) The mechanical properties of NMPs are influenced considerably by their microstructures (geometry, topology, size, *etc.*). However, based on the current MD or KMC simulation, it is hard to predict the microstructure of practical NMPs from the beginning of the dealloying process. Large-scale (both spatial and temporal) computational frameworks which can capture the details of the atomic scale should be developed. Moreover, the macroscopic surface of NPMs is very rough, which makes for an ultrahigh frictional coefficient. Exploring the mechanism of the frictional behavior may provide guidelines to design optimal-performance NPMs.
(5) Considering the complexity of the structures, simulations should also capture the plastic deformation localization at the weakest point of NMPs and the microscale heterogeneity of the structures. Besides, the chemical catalysis, thermal and electrical conductivity, and fluid transfer of NMPs are important for their potential applications, and these properties should be studied in future simulations.
(6) In the future, multi-scale computational simulation methods should be further developed to capture the hierarchical structures of NMPs to predict the physical and mechanical properties from the bottom level. The chemical and electrical properties are also very important in applications for NPMs, such as catalysts, chemical adsorption, sensors, *etc.* Further computational simulations should also describe these properties of NPMs. Besides, the NMPs could be polycrystalline and work in multiple fields (e.g., electromagnetic field, chemical field, *etc.*) which should also be considered in computational simulations.

## References

[1] Tappan B.C., Steiner S.A., Luther E.P. (2010). Nanoporous metal foams. Angew. Chem. Int. Ed..

[2] Dixon M.C., Daniel T.A., Hieda M., Smilgies D.M., Chan M.H.W., Allara D.L. (2007). Preparation, structure, and optical properties of nanoporous gold thin films. Langmuir.

[3] Wittstock A., Biener J., Erlebacher J., Bäumer M. (2012). Nanoporous Gold: From an Ancient Technology to a High-Tech Material.

[4] Ding Y., Chen M.W. (2009). Nanoporous metals for catalytic and optical applications. MRS Bull..

[5] Wittstock A., Biener J., Bäumer M. (2010). Nanoporous gold: A new material for catalytic and sensor applications. Phys. Chem. Chem. Phys..

[6] Xiao X., Ulstrup J., Li H., Wang M., Zhang J., Si P. (2014). Nanoporous gold assembly of glucose oxidase for electrochemical biosensing. Electrochim. Acta.

[7] Ruffato G., Romanato F., Garoli D., Cattarin S. (2011). Nanoporous gold plasmonic structures for sensing applications. Opt. Express.

[8] Hakamada M., Mabuchi M. (2013). Fabrication, microstructure, and properties of nanoporousPd, Ni, and their alloys by dealloying. Crit. Rev. Solid State.

[9] Weissmüller J., Newman R.C., Jin H.J., Hodge A.M., Kysar J.W. (2009). Nanoporous metals by alloy corrosion: Formation and mechanical properties. MRS Bull..

[10] Kramer D., Viswanath R.N., Weissmüller J. (2004). Surface-stress induced macroscopic bending of nanoporous gold cantilevers. Nano Lett..

[11] Jin H.J., Weissmüller J. (2011). A material with electrically tunable strength and flow stress. Science.

[12] Fujita T., Okada H., Koyama K., Watanabe K., Maekawa S., Chen M.W. (2008). Unusually small electrical resistance of three-dimensional nanoporous gold in external magnetic fields. Phys. Rev. Lett..

[13] Zhang L., Chang H., Hirata A., Wu H., Xue Q.K., Chen M.W. (2013). Nanoporous gold based optical sensor for sub-ppt detection of mercury ions. ACS Nano.

[14] Li D., Zhu Y., Wang H., Ding Y. (2013). Nanoporous gold as an active low temperature catalyst toward CO oxidation in hydrogen-rich stream. Sci. Rep. UK.

[15] Kashefi-Kheyrabadi L., Noori A., Mehrgardi M.A., Tiwari A., Turner A.P.F. (2014). Biosensors Nanotechnology.

[16] Xu Q. (2013). Nanoporous Materials: Synthesis and Applications.

[17] Seker E., Reed M.L., Begley M.R. (2009). Nanoporous gold: Fabrication, characterization, and applications. Materials.

[18] Biener J., Wittstock A., Baumann T.F., Weissmüller J., Bäumer M., Hamza A.V. (2009). Surface chemistry in nanoscale materials. Materials.

[19] Kertis F., Snyder J., Govada L., Khurshid S., Chayen N., Erlebacher J. (2010). Structure/processing relationships in the fabrication of nanoporous gold. JOM.

[20] Zheng L.T., Wei Y.L., Gong H.Q., Qian L. (2013). Application progress of nanoporous gold in analytical chemistry. Chin. J. Anal. Chem..

[21] Biener J., Wittstock A., Zepeda-Ruiz L.A., Biener M.M., Zielasek V., Kramer D., Viswanath R.N., Weissmüller J., Bäumer M., Hamza A.V. (2009). Surface-chemistry-driven actuation in nanoporous gold. Nat. Mater..

[22] Zhang Z.H., Wang Y., Qi Z., Zhuang W., Qin J., Frenzel J. (2009). Generalized fabrication of nanoporous metals (Au, Pd, Pt, Ag, and Cu) through chemical dealloying. J. Phys. Chem. C.

[23] Fujita T., Guan P., McKenna K., Lang X., Hirata A., Zhang L., Tokunaga T., Arai S., Yamamoto Y., Nobuo Tanaka N. (2012). Atomic origins of the high catalytic activity of nanoporous gold. Nat. Mater..

[24] Erlebacher J., Aziz M.J., Karma A., Dimitrov N., Sieradzki K. (2001). Evolution of nanoporosity in dealloying. Nature.

[25] Hyun S., Koo E. (2013). Scale-and shape-dependent transport property of nanoporous materials. J. Appl. Phys..

[26] Crowson D.A., Farkas D., Corcoran S.G. (2009). Mechanical stability of nanoporous metals with small ligament sizes. Scripta Mater..

[27] Curtarolo S., Setyawan W., Wang S., Xue J., Yang K., Taylor R.H., Nelson L.J., Hart G.L.W., Sanvito S., Buongiorno Nardelli M. (2012). AFLOWLIB.ORG: A distributed materials properties repository from high-throughput *ab initio* calculations. Comp. Mater. Sci..

[28] Epelbaum E., Krebs H., Lee D., Meißner U.-G. (2011). *Ab initio* calculation of the Hoyle State. Phys. Rev. Lett..

[29] Nazarov R., Hickel T., Neugebauer J. (2014). *Ab initio* study of H-vacancy interactions in fcc metals: Implications for the formation of superabundant vacancies. Phys. Rev. B.

[30] Jansen G.R., Engel J., Hagen G., Navratil P., Signoracci A. (2014). *Ab initio* coupled-cluster effective interactions for the shell model: Application to neutron-rich oxygen and carbon isotopes. Phys. Rev. Lett..

[31] Marx D., Hutter J. (2009). Ab Initio Molecular Dynamics: Basic Theory and Advanced Methods.

[32] Schaeffer H.F. (1984). Quantum Chemistry: The Development of Ab Initio Methods in Molecular Electronic Structure Theory.

[33] Arita R., Kuneš J., Kozhevnikov A.V., Equiluz A.G., Imada M. (2012). Ab initio studies on the interplay between spin-orbit interaction and Coulomb correlation in Sr_2_Ir_4_ and Ba_2_IrO_4_. Phys. Rev. Lett..

[34] Ohno K., Esfarjani K., Kawazoe Y. (1999). Computational Materials Science: From Ab Initio to Monte Carlo Methods.

[35] Marx D., Hutter J. (2000). Ab initio molecular dynamics: Theory and Implementation. Modern Methods and Algorithms of Quantum Chemistry.

[36] Saane S.S.R., Mangipudi K.R., Loos K.U., de Hosson J.T.M., Onck P.R. (2014). Multiscale modeling of charge-induced deformation of nanoporous gold structures. J. Mech. Phys. Solids.

[37] Liu Y.L., Xu Z.P. (2014). Multimodal and self-healable interfaces enable strong and tough grapheme-derived materials. J. Mech. Phys. Solids.

[38] Sun X.Y., Li Q., Gu Y., Feng X.Q. (2013). Mechanical properties of bioinspired bicontinuous nanocomposites. Comp. Mater. Sci..

[39] Liu Y.L., Chen X. (2014). Mechanical properties of nanoporousgraphene membrane. J. Appl. Phys..

[40] Allen M.P. (2004). Introduction to Molecular Dynamics Simulation. Computational Soft Matter: From Synthetic Polymers to Proteins.

[41] Wang C.C., Mao Y.W., Shan Z.W., Dao M., Li J., Sun J., Ma E., Suresh S. (2013). Real-time, high-resolution study of nanocrystallization and fatigue cracking in a cyclically strained metallic glass. Proc. Natl. Acad. Sci. USA.

[42] Weinberger C.R., Cai W. (2010). Plasticity of metal wires in torsion: Molecular dynamics and dislocation dynamics simulations. J. Mech. Phys. Solids.

[43] Rao S.I., Dimiduk D.M., El-Awady J.A., Parthasarathy T.A., Uchic M.D., Woodward C. (2013). Spontaneous athermal cross-slip nucleation at screw dislocation intersections in FCC metals and L1 2 intermetallics investigated via atomistic simulations. Philos. Mag..

[44] Higginbotham A., Suggit M.J., Bringa E.M., Erhart P., Hawreliak J.A., Mogni G., Park N., Remington B.A., Wark J.S. (2013). Molecular dynamics simulations of shock-induced deformation twinning of a body-centered-cubic metal. Phys. Rev. B.

[45] Ravelo R., Germann T.C., Guerrero O., An Q., Holian B.L. (2013). Shock-induced plasticity in tantalum single crystals: Interatomic potentials and large-scale molecular-dynamics simulations. Phys. Rev. B.

[46] Li X., Wei Y., Lu L., Lu K., Gao H. (2010). Dislocation nucleation governed softening and maximum strength in nano-twinned metals. Nature.

[47] Zhu T., Gao H. (2012). Plastic deformation mechanism in nanotwinned metals: An insight from molecular dynamics and mechanistic modeling. Scr. Mater..

[48] Rodriguez-Nieva J.F., Bringa E.M. (2013). Molecular dynamics and Monte Carlo simulations of the sputtering of a nanoporous solid. Nuclear Instrum. Methods B.

[49] Chen S., Liang J., Mo Y., Luo D., Jiang S. (2013). Onset of shadowing-dominated growth of Ag films in glancing angle deposition: Kinetic Monte Carlo simulation. Appl. Surf. Sci..

[50] Schulze T.P., Smereka P. (2012). Kinetic Monte Carlo simulation of heteroepitaxial growth: Wetting layers, quantum dots, capping, and nanorings. Phys. Rev. B.

[51] Castin N., Pascuet M.I., Malerba L. (2012). Mobility and stability of large vacancy and vacancy–copper clusters in iron: An atomistic kinetic Monte Carlo study. J. Nuclear Mater..

[52] Dholabhai P.P., Anwar S., Adams J.B., Crozier P., Sharma R. (2011). Kinetic lattice Monte Carlo model for oxygen vacancy diffusion in praseodymium doped ceria: Applications to materials design. J. Solid State Chem..

[53] Molnar D., Mukherjee R., Choudhury A., Mora A., Binkele P., Selzer M., Nestler B., Schmauder S. (2012). Multiscale simulations on the coarsening of Cu-rich precipitates in α-Fe using kinetic Monte Carlo, molecular dynamics and phase-field simulations. Acta Mater..

[54] Zhang X., Gao W., Bellon P., Averback R.S., Zuo J.M. (2014). A kinetic Monte Carlo study of coarsening resistance of novel core/shell precipitates. Acta Mater..

[55] Erlebacher J. (2004). An atomistic description of dealloying porosity evolution, the critical potential, and rate-limiting behavior. J. Electrochem. Soc..

[56] Zinchenko O., De Raedt H.A., Detsi E., Onck P.R., de Hosson J.T.M. (2013). Nanoporous gold formation by dealloying: A Metropolis Monte Carlo study. Comput. Phys. Commun..

[57] Erlebacher J., McCue I. (2012). Geometric characterization of nanoporous metals. Acta Mater..

[58] Wang J., Lam D.C. (2009). Model and analysis of size-stiffening in nanoporouscellular solids. J. Mater. Sci..

[59] Huang Y. (1991). A User-Material Subroutine Incorporating Single Crystal Plasticity in the ABAQUS Finite Element Program, Mech Report 178.

[60] Lee D., Wei X., Zhao M., Chen X., Jun S.C., Hone J., Kysar J.W. (2007). Plastic deformation in nanoscale gold single crystals and open-celled nanoporous gold. Model. Simul. Mater. Sci..

[61] Zhu H.X., Zhang H.C., You J.F., Kennedy D., Wang Z.B., Fan T.X., Zhang D. (2014). The elastic and geometrical properties of micro-and nano-structured hierarchical random irregular honeycombs. J. Mater. Sci..

[62] Fujita T., Qian L.H., Inoke K., Erlebacher J., Chen M.W. (2008). Three-dimensional morphology of nanoporous gold. Appl. Phys. Lett..

[63] Newman R.C., Corcoran S.G., Erlebacher J., Aziz M.J., Sieradzki K. (1999). Alloy corrosion. MRS Bull..

[64] Pugh D.V. (2003). Nanoporous Platinum. Ph.D. Thesis.

[65] Chan J.W., Hilliard J.E. (1958). Free energy of a non-uniform system I. interfacial free energy. J. Chem. Phys..

[66] Rashed J. (2009). Coarsening Dynamics for the Cahn-Hilliard Equation. Master’s Thesis.

[67] Rugolo J., Erlebacher J., Sieradzki K. (2006). Length scales in alloy dissolution and measurement of absolute interfacial free energy. Nat. Mater..

[68] Artymowicz D.M., Erlebacher J., Newman R.C. (2009). Relationship between the parting limit for de-alloying and a particular geometric high-density site percolation threshold. Philos. Mag..

[69] Santiago-Cortés C.U., Mejía-Mendoza L.M., Valladares R.M., Valladares A., Valladares A.A. (2012). Computational alternatives to generate amorphous nanoporous structures using *ab initio* molecular dynamics. J. Non-Cryst. Solids.

[70] Shruti M.B. (2010). Statistical Analysis of Factors Affecting Nanoporous Gold and its Sensitivity in Comparison with Bulk Gold. Master’s Thesis.

[71] Chen-Wiegart Y.K., Wang S., McNulty I., Dunand D.C. (2013). Effect of Ag-Au composition and acid concentration on dealloying front velocity and cracking during nanoporous gold formation. Acta Mater..

[72] Adisoejoso J., Tahara K., Lei S., Szabelski P., Rzysko W., Inukai K., Blunt M.O., Tobe Y., De Feyter S. (2012). One building block, two different nanoporousself-assembled monolayers: A combined STM and Monte Carlo study. ACS Nano.

[73] Kolluri K., Demkowicz M.J. (2011). Coarsening by network restructuring in model nanoporous gold. Acta Mater..

[74] Erlebacher J. (2011). Mechanism of coarsening and bubble formation in high-genus nanoporous metals. Phys. Rev. Lett..

[75] Detsi E., de Jong E., Zinchenko A., Vuković Z., Vuković I., Punzhin S., Loos K., Ten Brinke G., de Raedt H.A., Onck P.R. (2011). On the specific surface area of nanoporous materials. Acta Mater..

[76] Xia R., Feng X.Q., Wang G.F. (2011). Effective elastic properties of nanoporous materials with hierarchical structure. Acta Mater..

[77] Crowson D.A., Farkas D., Corcoran S.G. (2007). Geometric relaxation of nanoporous metals: The role of surface relaxation. Scripta Mater..

[78] Weissmüller J., Duan H.L., Farkas D. (2010). Deformation of solids with nanoscale pores by the action of capillary forces. Acta Mater..

[79] Abdolrahim N., Bahr D.F., Revard B., Reilly C., Ye J., Balk T.J., Zbib H.M. (2013). The mechanical response of core-shell structures for nanoporous metallic materials. Philos. Mag..

[80] Cantrell C. (2006). Molecular Dynamics Simulation of Mechanical Behavior of Nanoporous Copper Foams. Bachelor’s Thesis.

[81] Ruestes C.J., Bringa E.M., Stukowski A., Rodríguez Nieva J.F., Tang Y., Meyers M.A. (2014). Plastic deformation of a porous bcc metal containing nanometer sized voids. Comp. Mater. Sci..

[82] Ruestes C.J., Bringa E.M., Stukowski A., Rodríguez Nieva J.F., Bertolino G., Tang Y., Meyers M.A. (2013). Atomistic simulation of the mechanical response of a nanoporous body-centered cubic metal. Scr. Mater..

[83] Erhart P., Bringa E.M., Kumar M., Albe K. (2005). Atomistic mechanism of shock-induced void collapse in nanoporous metals. Phys. Rev. B.

[84] To A.C., Tao J., Kirca M., Schalk L. (2011). Ligament and joint sizes govern softening in nanoporous aluminum. Appl. Phys. Lett..

[85] Giri A. (2012). Molecular Dynamics Simulations of the Mechanical Deformation of Nanoporous Gold. Bachelor’s Thesis.

[86] Giri A., Tao J., Wang L., Kirca M., To A.C. (2013). Compressive behavior and deformation mechanism of nanoporous open-cell foam with ultrathin ligaments. J. Nanomech. Micromech..

[87] Sun X.Y., Xu G.K., Li X., Feng X.Q., Gao H. (2013). Mechanical properties and scaling laws of nanoporous gold. J. Appl. Phys..

[88] Farkas D., Caro A., Bringa E., Crowson D. (2013). Mechanical response of nanoporous gold. Acta Mater..

[89] Biener J., Hodge A.M., Hayes J.R., Volkert C.A., Zepeda-Ruiz L.A., Hamza A.V., Abraham F.F. (2006). Size effects on the mechancial behavior of nanoporous Au. Nano Lett..

[90] Xia R., Wang J.L., Wang R., Zhang X., Feng X.Q., Ding Y. (2010). Correlation of the thermal and electrical conductivities of nanoporous gold. Nanotechnology.

[91] Frenkel A.I., Vasić R., Dukesz B., Li D., Chen M.W., Zhang L., Fujita T. (2012). Thermal properties of nanoporous gold. Phys. Rev. B.

[92] Cheng I.C., Hodge A.M. (2012). Morphology, oxidation, and mechanical behavior of nanoporous Cu foams. Adv. Eng. Mater..

[93] Wang J.L., Xia R., Zhu J., Ding Y., Zhang X., Chen Y. (2012). Effect of thermal coarsening on the themal conductivity of nanoporous gold. J. Mater. Sci..

[94] Seker E., Gaskins J.T., Bart-Smith H., Zhu J., Reed M.L., Zangari G., Kelly R., Begley M.R. (2008). The effects of annealing prior to dealloying on the mechanical properties of nanoporous gold microbeams. Acta Mater..

[95] Luke J.R., Tien C.L. (2004). Molecular dynamics simulation of thermal conduction in nanoporous thin films. Microscale Therm. Eng..

[96] Bera C., Mingo N., Volz S. (2010). Marked effects of alloying on the thermal conductivity of nanoporous materials. Phys. Rev. Lett..

[97] Sardana N., Birr T., Schlenker S., Reinhardt C., Schilling J. (2014). Surface plasmons on ordered and bi-continuous spongy nanoporous gold. New J. Phys..

[98] Li R., Liu X.J., Wang H., Wu X., Chu X.M., Lu Z.P. (2014). Nanoporous silver with tunable pore characteristics and superior surface enhanced Raman scattering. Corros. Sci..

[99] Qi J., Motwani P., Gheewala M., Brennan C., Wolfe J.C., Shih W.-C. (2013). Surface-enhanced Raman spectroscopy with monolithic nanoporous gold disk substrates. Nanoscale.

[100] Detsi E., Salverda M., Onck P.R., De Hosson J.T.M. (2014). On the localized sufaceplasmon resonance modes in nanoporous gold films. J. Appl. Phys..

[101] Bosman M., Anstis G.R., Keast V.J., Clarke J.D., Cortie M.B. (2011). Light splitting in nanoporous gold and silver. ACS Nano.

[102] Fu Y., Zhang J., Nowaczyk K., Lakowicz J.R. (2013). Enhanced single molecule fluorescence and reduced observation volumes on nanoporous gold (NPG) films. Chem. Commun..

[103] Fu C., Gu Y., Wu Z., Wang Y., Xu S., Xu W. (2014). Surface-enhanced Raman scattering (SERS) biosensing based on nanoporous dielectric waveguide resonance. Sens. Actuators B Chem..

[104] Zhao F., Zeng J., Parvez A.M., Sun P., Qi J., Motwani P., Gheewala M., Li C., Paterson A., Strych U. (2014). Monolithic NPG nanoparticles with large surface area, tunable plasmonics, and high-density internal hot-spots. Nanoscale.

[105] Hakamada M., Hirashima F., Takahashi M., Nakazawa T., Mabuchi M. (2011). Large-strain-induced magnetic properties of Co electrodeposited on nanoporous Au. J. Appl. Phys..

[106] Wittstock A., Wichmann A., Bäumer M. (2012). Nanoporous gold as a platform for a building block catalyst. ACS Catal..

[107] Xu C., Su J., Xu X., Liu P., Zhao H., Tian F., Ding Y. (2007). Low temperature CO oxidation over unsupported nanoporous gold. J. Am. Chem. Soc..

[108] Beyerlein I.J., Caro A., Demkowicz M.J., Mara N.A., Misra A., Uberuaga B.P. (2013). Radiation damage tolerant nanomaterials. Mater. Today.

[109] Bringa E.M., Monk J.D., Caro A., Misra A., Zepeda-Ruiz L., Duchaineau M., Nastasi M., Picraux S.T., Wang Y.Q., Farkas D. (2011). Are nanoporous materials radiation resistant?. Nano Lett..

[110] Fu E.G., Caro M., Zepeda-Ruiz L.A., Wang Y.Q., Baldwin K., Nastasi M., Bringa E., Nastasi M., Caro A. (2012). Surface effects on the radiation response of nanoporous Au foams. Appl. Phys. Lett..

[111] Sun C., Bufford D., Chen Y., Kirk M.A., Wang Y.Q., Li M., Wang H., Maloy S.A., Zhang X. (2014). *In situ* study of defect migration kinetics in nanoporous Ag with enhanced radiation tolerance. Sci. Rep..

[112] Biener M.M., Biener J., Wichmann A., Wittstock A., Baumann T.F., Bäumer M., Hamza A.V. (2011). ALD functionalized nanoporous gold: Thermal stability, mechanical properties, and catalytic activity. Nano Lett..

[113] Hakamada M., Mabuchi M. (2006). Nanoporous gold prism microassembly through a self-organizing route. Nano Lett..

